# 7-Chloro-4-[(*E*)-2-(2,5-dimeth­oxy­benzyl­idene)hydrazin-1-yl]quinoline

**DOI:** 10.1107/S1600536812012871

**Published:** 2012-03-31

**Authors:** Marcus V. N. de Souza, Marcelle de Lima Ferreira, Solange M. S. V. Wardell, Edward R. T. Tiekink, James L. Wardell

**Affiliations:** a Instituto de Tecnologia em Fármacos–Farmanguinhos, FioCruz–Fundação Oswaldo Cruz, R. Sizenando Nabuco, 100, Manguinhos, 21041-250 Rio de Janeiro, RJ, Brazil; b CHEMSOL, 1 Harcourt Road, Aberdeen AB15 5NY, Scotland; c Department of Chemistry, University of Malaya, 50603 Kuala Lumpur, Malaysia; d Centro de Desenvolvimento Tecnológico em Saúde (CDTS), Fundação Oswaldo Cruz (FIOCRUZ), Casa Amarela, Campus de Manguinhos, Av. Brasil 4365, 21040-900 Rio de Janeiro, RJ, Brazil

## Abstract

In the nearly planar title compound (r.m.s. deviation for the 24 non-H atoms = 0.064 Å), C_18_H_16_ClN_3_O_2_, the conformation about the N=C bond is *E*. Supra­molecular chains propagated by glide symmetry along [001] are found in the crystal packing. These are sustained by N—H⋯N hydrogen bonds with the quinoline N atom being the acceptor. The chains are connected into a three-dimensional architecture by π–π inter­actions involving all three aromatic rings [centroid–centroid distances = 3.5650 (9)–3.6264 (9) Å].

## Related literature

For the biological activity, including anti-tubercular and anti-tumour activity, of compounds containing the quinolinyl nucleus, see: de Souza *et al.* (2009 ▶); Candea *et al.* (2009 ▶); Montenegro *et al.* (2011 ▶, 2012 ▶). For related structures, see: Howie *et al.* (2010 ▶); de Souza *et al.* (2010 ▶); de Lima Ferreira *et al.* (2010 ▶). For the synthesis, see: Montenegro *et al.* (2012 ▶).
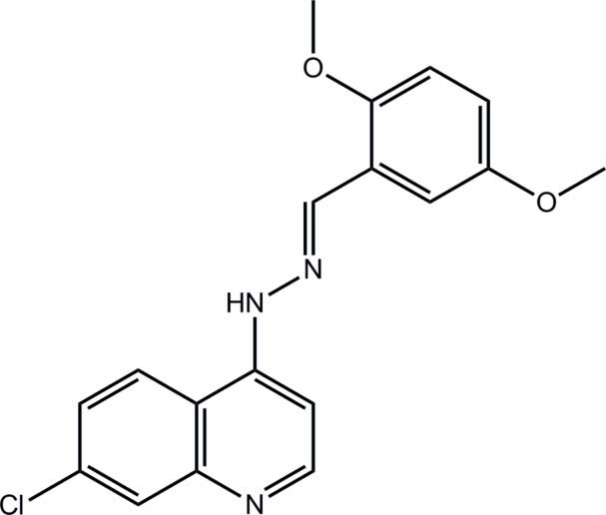


## Experimental

### 

#### Crystal data

C_18_H_16_ClN_3_O_2_
*M* *_r_* = 341.79
Monoclinic, 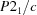

*a* = 10.5183 (2) Å
*b* = 12.9132 (3) Å
*c* = 12.9861 (2) Å
β = 112.723 (2)°
*V* = 1626.93 (5) Å^3^
*Z* = 4
Mo *K*α radiation
μ = 0.25 mm^−1^
*T* = 120 K
0.32 × 0.20 × 0.15 mm

#### Data collection

Bruker–Nonius Roper CCD camera on a κ-goniostat diffractometer
Absorption correction: multi-scan (*SADABS*; Sheldrick, 2007 ▶) *T*_min_ = 0.652, *T*_max_ = 0.746
20405 measured reflections
3723 independent reflections
3067 reflections with *I* > 2σ(*I*)
*R*_int_ = 0.049

#### Refinement

*R*[*F* ^2^ > 2σ(*F* ^2^)] = 0.041
*wR*(*F* ^2^) = 0.107
*S* = 1.03
3723 reflections
222 parameters
1 restraint
H atoms treated by a mixture of independent and constrained refinement
Δρ_max_ = 0.33 e Å^−3^
Δρ_min_ = −0.33 e Å^−3^

### 

Data collection: *COLLECT* (Hooft, 1998 ▶); cell refinement: *DENZO* (Otwinowski & Minor, 1997 ▶) and *COLLECT*; data reduction: *DENZO* and *COLLECT*; program(s) used to solve structure: *SHELXS97* (Sheldrick, 2008 ▶); program(s) used to refine structure: *SHELXL97* (Sheldrick, 2008 ▶); molecular graphics: *ORTEP-3* (Farrugia, 1997 ▶) and *DIAMOND* (Brandenburg, 2006 ▶); software used to prepare material for publication: *publCIF* (Westrip, 2010 ▶).

## Supplementary Material

Crystal structure: contains datablock(s) global, I. DOI: 10.1107/S1600536812012871/xu5494sup1.cif

Structure factors: contains datablock(s) I. DOI: 10.1107/S1600536812012871/xu5494Isup2.hkl

Supplementary material file. DOI: 10.1107/S1600536812012871/xu5494Isup3.cml

Additional supplementary materials:  crystallographic information; 3D view; checkCIF report

## Figures and Tables

**Table 1:** Hydrogen-bond geometry (Å, °)

*D*—H⋯*A*	*D*—H	H⋯*A*	*D*⋯*A*	*D*—H⋯*A*
N2—H2n⋯N1^i^	0.88 (1)	2.19 (1)	3.0572 (17)	167 (2)

Symmetry code: (i) 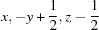.

**Fig. 1.**
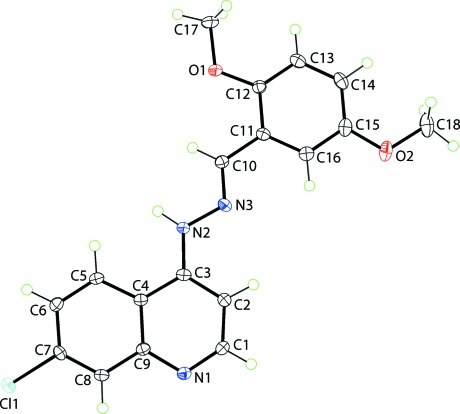
The molecular structure showing the atom-labelling scheme and displacement ellipsoids at the 50% probability level.

**Fig. 2.**
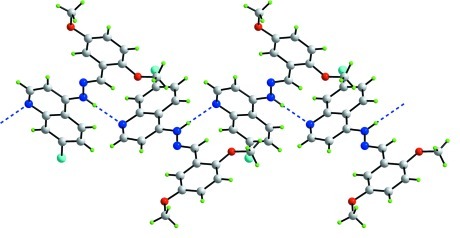
A view of the supramolecular chain along [001] in (I). The N—H···N hydrogen bonds are shown as blue dashed lines.

**Fig. 3.**
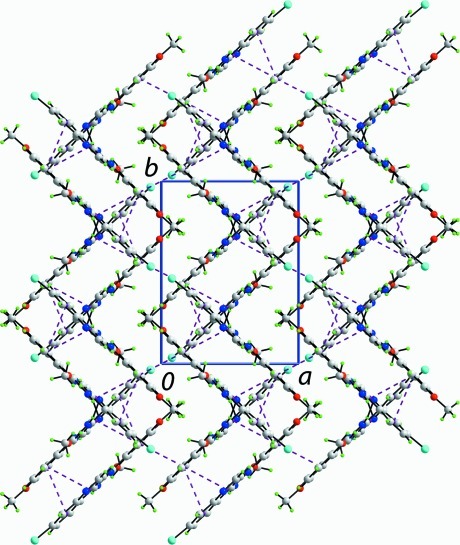
A view in projection down the c axis of unit-cell contents of (I). The N—H···N and π···π interactions are shown as blue and purple dashed lines, respectively.

## supplementary crystallographic information

### Comment

The quinoline nucleus is an important moiety found in various synthetic and natural products with a wide range of pharmacological activities (de Souza *et al.*, 2009), including anti-tubercular (Candea *et al.*, 2009) and anti-tumour (Montenegro *et al.*, 2012) activities. Among the derivatives studied have been arylaldehyde 7-chloroquinoline-4-hydrazones (Candea *et al.*, 2009; Montenegro *et al.*, 2011). Some crystal structures of the these hydrazones, including related methoxy-substituted derivatives have been reported (Howie *et al.*, 2010; de Souza *et al.*, 2010; de Lima Ferreira *et al.*, 2010). We now wish to report the crystal structure of the title compound, (I).

In (I), Fig. 1, the entire molecule is planar with the r.m.s. deviation of all 24 non-hydrogen atoms being 0.064 Å. The maximum deviations from the least-squares plane are 0.108 (2) for the C5 atom and -0.165 (1) Å for the Cl1 atom. The conformation about the N3═C10 bond [1.2834 (18) Å] is *E*.

The most prominent feature of the crystal packing is the formation of supramolecular chains *via* N—H···N hydrogen bonds with the quinolinyl-N atom being the acceptor, Table 1. The chains a propagated by glide symmetry along the *c* axis, Fig. 2. Molecules are consolidated into a three-dimensional architecture by π—π interactions whereby the dimethoxybenzene ring interacts with both components of the quinolinyl residue along with symmetry related dimethoxybenzene rings [centroid(dimethoxybenzene)···centroid(NC_5_)^i^; (C_6_)^i^; (dimethoxybenzene)^ii^ = 3.5650 (9), 3.6264 (9) and 3.5872 (9) Å, with angles of inclination = 2.36 (7) 4.20 (7) and 0° for symmetry operations *i*: 1 - *x*, -*y*, 1 - *z* and *ii*: 2 - *x*, -*y*, 1 - *z*]. The π—π interactions between the dimethoxybenzene and quinolinyl residues lead to zigzag layers in the *bc* plane and the π(dimethoxybenzene)···π(dimethoxybenzene) interactions link these layers along the *a* axis, Fig. 3.

### Experimental

The compound was prepared from 7-chloro-4-quinolinylhydrazone with 2,5-dimethoxybenzaldehyde (Montenegro *et al.*, 2012). The crystals used in the structure determination were grown from an ethanol solution of the compound.

### Refinement

The C-bound H atoms were geometrically placed (C—H = 0.95–0.98 Å) and refined as riding with *U*_iso_(H) = 1.2–1.5*U*_eq_(C). The N-bound H-atom was located in a difference Fourier map and refined with a N—H distance = 0.88±0.01 Å, and with *U*_iso_(H) = 1.2*U*_eq_(N). Owing to poor agreement, the (1 0 2) and (2 3 0) reflections were omitted from the final cycles of refinement.

### Crystal data

**Table:**

C_18_H_16_ClN_3_O_2_	*F*(000) = 712
*M**_r_* = 341.79	*D*_x_ = 1.395 Mg m^−^^3^
Monoclinic, *P*2_1_/*c*	Mo *K*α radiation, λ = 0.71073 Å
Hall symbol: -P 2ybc	Cell parameters from 8419 reflections
*a* = 10.5183 (2) Å	θ = 2.9–27.5°
*b* = 12.9132 (3) Å	µ = 0.25 mm^−^^1^
*c* = 12.9861 (2) Å	*T* = 120 K
β = 112.723 (2)°	Prism, yellow
*V* = 1626.93 (5) Å^3^	0.32 × 0.20 × 0.15 mm
*Z* = 4	

### Data collection

**Table:**

Bruker–Nonius Roper CCD camera on a κ-goniostat diffractometer	3723 independent reflections
Radiation source: Bruker–Nonius FR591 rotating anode	3067 reflections with *I* > 2σ(*I*)
Graphite monochromator	*R*_int_ = 0.049
Detector resolution: 9.091 pixels mm^-1^	θ_max_ = 27.5°, θ_min_ = 3.2°
φ and ω scans	*h* = −13→13
Absorption correction: multi-scan (*SADABS*; Sheldrick, 2007)	*k* = −15→16
*T*_min_ = 0.652, *T*_max_ = 0.746	*l* = −16→16
20405 measured reflections	

### Refinement

**Table:**

Refinement on *F*^2^	Primary atom site location: structure-invariant direct methods
Least-squares matrix: full	Secondary atom site location: difference Fourier map
*R*[*F*^2^ > 2σ(*F*^2^)] = 0.041	Hydrogen site location: inferred from neighbouring sites
*wR*(*F*^2^) = 0.107	H atoms treated by a mixture of independent and constrained refinement
*S* = 1.03	*w* = 1/[σ^2^(*F*_o_^2^) + (0.0516*P*)^2^ + 0.6611*P*] where *P* = (*F*_o_^2^ + 2*F*_c_^2^)/3
3723 reflections	(Δ/σ)_max_ = 0.001
222 parameters	Δρ_max_ = 0.33 e Å^−^^3^
1 restraint	Δρ_min_ = −0.33 e Å^−^^3^

### Special details

**Table:**

Geometry. All s.u.'s (except the s.u. in the dihedral angle between two l.s. planes) are estimated using the full covariance matrix. The cell s.u.'s are taken into account individually in the estimation of s.u.'s in distances, angles and torsion angles; correlations between s.u.'s in cell parameters are only used when they are defined by crystal symmetry. An approximate (isotropic) treatment of cell s.u.'s is used for estimating s.u.'s involving l.s. planes.
Refinement. Refinement of *F*^2^ against ALL reflections. The weighted *R*-factor *wR* and goodness of fit *S* are based on *F*^2^, conventional *R*-factors *R* are based on *F*, with *F* set to zero for negative *F*^2^. The threshold expression of *F*^2^ > 2σ(*F*^2^) is used only for calculating *R*-factors(gt) *etc*. and is not relevant to the choice of reflections for refinement. *R*-factors based on *F*^2^ are statistically about twice as large as those based on *F*, and *R*- factors based on ALL data will be even larger.

### Fractional atomic coordinates and isotropic or equivalent isotropic displacement parameters (Å^2^)

**Table:**

	*x*	*y*	*z*	*U*_iso_*/*U*_eq_	
Cl1	0.07757 (4)	0.46710 (3)	0.65860 (3)	0.02240 (13)	
O1	0.67832 (11)	0.06456 (8)	0.25193 (8)	0.0200 (2)	
O2	0.98754 (12)	−0.18234 (9)	0.60966 (10)	0.0273 (3)	
N1	0.45113 (13)	0.19900 (10)	0.83352 (10)	0.0169 (3)	
N2	0.53424 (13)	0.16602 (10)	0.54269 (10)	0.0169 (3)	
H2N	0.4970 (16)	0.2057 (11)	0.4831 (10)	0.020*	
N3	0.63038 (13)	0.09359 (10)	0.54186 (10)	0.0174 (3)	
C1	0.54351 (15)	0.13277 (12)	0.82563 (12)	0.0173 (3)	
H1	0.5927	0.0921	0.8897	0.021*	
C2	0.57480 (15)	0.11751 (12)	0.73189 (12)	0.0172 (3)	
H2	0.6407	0.0669	0.7327	0.021*	
C3	0.50855 (15)	0.17718 (11)	0.63725 (12)	0.0143 (3)	
C4	0.40798 (14)	0.25126 (11)	0.64014 (11)	0.0135 (3)	
C5	0.33252 (15)	0.31670 (11)	0.54965 (12)	0.0157 (3)	
H5	0.3502	0.3143	0.4833	0.019*	
C6	0.23425 (15)	0.38355 (11)	0.55596 (12)	0.0168 (3)	
H6	0.1850	0.4277	0.4950	0.020*	
C7	0.20770 (15)	0.38559 (11)	0.65392 (12)	0.0155 (3)	
C8	0.27876 (15)	0.32511 (11)	0.74402 (12)	0.0158 (3)	
H8	0.2589	0.3287	0.8094	0.019*	
C9	0.38202 (14)	0.25713 (11)	0.73985 (12)	0.0142 (3)	
C10	0.64283 (15)	0.08142 (11)	0.44812 (12)	0.0157 (3)	
H10	0.5869	0.1209	0.3851	0.019*	
C11	0.74242 (15)	0.00736 (11)	0.43720 (12)	0.0145 (3)	
C12	0.75975 (15)	−0.00070 (11)	0.33516 (12)	0.0156 (3)	
C13	0.85456 (16)	−0.07004 (12)	0.32490 (13)	0.0194 (3)	
H13	0.8663	−0.0752	0.2561	0.023*	
C14	0.93308 (16)	−0.13246 (12)	0.41476 (13)	0.0203 (3)	
H14	0.9977	−0.1800	0.4070	0.024*	
C15	0.91658 (15)	−0.12495 (12)	0.51558 (13)	0.0195 (3)	
C16	0.82194 (15)	−0.05485 (11)	0.52614 (13)	0.0172 (3)	
H16	0.8114	−0.0494	0.5954	0.021*	
C17	0.7013 (2)	0.06604 (15)	0.15086 (14)	0.0303 (4)	
H17A	0.7981	0.0827	0.1674	0.045*	
H17B	0.6421	0.1185	0.1005	0.045*	
H17C	0.6796	−0.0021	0.1151	0.045*	
C18	1.09064 (17)	−0.25167 (13)	0.60485 (16)	0.0306 (4)	
H18A	1.0479	−0.3035	0.5466	0.046*	
H18B	1.1349	−0.2864	0.6771	0.046*	
H18C	1.1599	−0.2127	0.5876	0.046*	

### Atomic displacement parameters (Å^2^)

**Table:**

	*U*^11^	*U*^22^	*U*^33^	*U*^12^	*U*^13^	*U*^23^
Cl1	0.0225 (2)	0.0242 (2)	0.0220 (2)	0.00878 (15)	0.01015 (16)	0.00116 (15)
O1	0.0247 (6)	0.0234 (6)	0.0150 (5)	0.0069 (5)	0.0111 (5)	0.0024 (4)
O2	0.0263 (6)	0.0217 (6)	0.0280 (6)	0.0076 (5)	0.0040 (5)	0.0068 (5)
N1	0.0197 (6)	0.0178 (6)	0.0140 (6)	0.0014 (5)	0.0073 (5)	0.0000 (5)
N2	0.0197 (7)	0.0191 (7)	0.0141 (6)	0.0052 (5)	0.0091 (5)	0.0022 (5)
N3	0.0185 (6)	0.0176 (6)	0.0188 (6)	0.0031 (5)	0.0100 (5)	−0.0005 (5)
C1	0.0189 (8)	0.0183 (7)	0.0143 (7)	0.0015 (6)	0.0057 (6)	0.0024 (6)
C2	0.0175 (7)	0.0184 (7)	0.0161 (7)	0.0028 (6)	0.0067 (6)	0.0002 (6)
C3	0.0148 (7)	0.0152 (7)	0.0139 (7)	−0.0041 (5)	0.0068 (6)	−0.0031 (5)
C4	0.0141 (7)	0.0143 (7)	0.0127 (7)	−0.0026 (5)	0.0057 (6)	−0.0017 (5)
C5	0.0183 (7)	0.0174 (7)	0.0128 (7)	−0.0011 (6)	0.0076 (6)	0.0005 (6)
C6	0.0188 (7)	0.0155 (7)	0.0157 (7)	0.0000 (6)	0.0063 (6)	0.0024 (6)
C7	0.0149 (7)	0.0127 (7)	0.0190 (7)	0.0007 (5)	0.0067 (6)	−0.0029 (6)
C8	0.0184 (7)	0.0171 (7)	0.0141 (7)	−0.0010 (6)	0.0085 (6)	−0.0019 (6)
C9	0.0157 (7)	0.0139 (7)	0.0125 (7)	−0.0025 (6)	0.0048 (5)	−0.0021 (5)
C10	0.0163 (7)	0.0164 (7)	0.0150 (7)	−0.0008 (6)	0.0067 (6)	0.0006 (6)
C11	0.0144 (7)	0.0139 (7)	0.0162 (7)	−0.0024 (5)	0.0072 (6)	−0.0017 (5)
C12	0.0166 (7)	0.0152 (7)	0.0154 (7)	−0.0007 (6)	0.0065 (6)	−0.0002 (5)
C13	0.0207 (8)	0.0188 (8)	0.0218 (8)	−0.0006 (6)	0.0113 (6)	−0.0039 (6)
C14	0.0171 (7)	0.0152 (7)	0.0296 (8)	0.0008 (6)	0.0101 (7)	−0.0029 (6)
C15	0.0165 (7)	0.0142 (7)	0.0237 (8)	−0.0015 (6)	0.0031 (6)	0.0012 (6)
C16	0.0179 (7)	0.0162 (7)	0.0176 (7)	−0.0029 (6)	0.0071 (6)	−0.0014 (6)
C17	0.0427 (11)	0.0363 (10)	0.0193 (8)	0.0131 (8)	0.0202 (8)	0.0059 (7)
C18	0.0236 (9)	0.0184 (8)	0.0431 (11)	0.0048 (7)	0.0053 (8)	0.0037 (7)

### Geometric parameters (Å, º)

**Table:**

Cl1—C7	1.7462 (15)	C6—H6	0.9500
O1—C12	1.3773 (18)	C7—C8	1.365 (2)
O1—C17	1.4235 (18)	C8—C9	1.414 (2)
O2—C15	1.3764 (18)	C8—H8	0.9500
O2—C18	1.426 (2)	C10—C11	1.465 (2)
N1—C1	1.3277 (19)	C10—H10	0.9500
N1—C9	1.3742 (18)	C11—C16	1.390 (2)
N2—C3	1.3631 (18)	C11—C12	1.409 (2)
N2—N3	1.3805 (17)	C12—C13	1.384 (2)
N2—H2N	0.884 (9)	C13—C14	1.396 (2)
N3—C10	1.2834 (18)	C13—H13	0.9500
C1—C2	1.392 (2)	C14—C15	1.389 (2)
C1—H1	0.9500	C14—H14	0.9500
C2—C3	1.389 (2)	C15—C16	1.391 (2)
C2—H2	0.9500	C16—H16	0.9500
C3—C4	1.437 (2)	C17—H17A	0.9800
C4—C5	1.416 (2)	C17—H17B	0.9800
C4—C9	1.4248 (19)	C17—H17C	0.9800
C5—C6	1.373 (2)	C18—H18A	0.9800
C5—H5	0.9500	C18—H18B	0.9800
C6—C7	1.404 (2)	C18—H18C	0.9800
			
C12—O1—C17	117.14 (12)	N3—C10—C11	120.69 (13)
C15—O2—C18	117.38 (13)	N3—C10—H10	119.7
C1—N1—C9	115.86 (12)	C11—C10—H10	119.7
C3—N2—N3	118.64 (12)	C16—C11—C12	118.92 (13)
C3—N2—H2N	123.5 (11)	C16—C11—C10	121.33 (13)
N3—N2—H2N	117.7 (11)	C12—C11—C10	119.74 (13)
C10—N3—N2	115.71 (12)	O1—C12—C13	124.95 (13)
N1—C1—C2	125.86 (13)	O1—C12—C11	115.19 (13)
N1—C1—H1	117.1	C13—C12—C11	119.85 (14)
C2—C1—H1	117.1	C12—C13—C14	120.57 (14)
C3—C2—C1	119.21 (13)	C12—C13—H13	119.7
C3—C2—H2	120.4	C14—C13—H13	119.7
C1—C2—H2	120.4	C15—C14—C13	119.91 (14)
N2—C3—C2	122.16 (13)	C15—C14—H14	120.0
N2—C3—C4	119.86 (13)	C13—C14—H14	120.0
C2—C3—C4	117.96 (13)	O2—C15—C14	125.22 (14)
C5—C4—C9	118.67 (13)	O2—C15—C16	115.23 (14)
C5—C4—C3	123.90 (13)	C14—C15—C16	119.55 (14)
C9—C4—C3	117.42 (12)	C11—C16—C15	121.20 (14)
C6—C5—C4	121.28 (13)	C11—C16—H16	119.4
C6—C5—H5	119.4	C15—C16—H16	119.4
C4—C5—H5	119.4	O1—C17—H17A	109.5
C5—C6—C7	118.92 (13)	O1—C17—H17B	109.5
C5—C6—H6	120.5	H17A—C17—H17B	109.5
C7—C6—H6	120.5	O1—C17—H17C	109.5
C8—C7—C6	122.10 (13)	H17A—C17—H17C	109.5
C8—C7—Cl1	119.49 (11)	H17B—C17—H17C	109.5
C6—C7—Cl1	118.41 (11)	O2—C18—H18A	109.5
C7—C8—C9	119.80 (13)	O2—C18—H18B	109.5
C7—C8—H8	120.1	H18A—C18—H18B	109.5
C9—C8—H8	120.1	O2—C18—H18C	109.5
N1—C9—C8	117.18 (12)	H18A—C18—H18C	109.5
N1—C9—C4	123.63 (13)	H18B—C18—H18C	109.5
C8—C9—C4	119.18 (13)		
			
C3—N2—N3—C10	175.16 (13)	C3—C4—C9—N1	2.5 (2)
C9—N1—C1—C2	−0.3 (2)	C5—C4—C9—C8	2.2 (2)
N1—C1—C2—C3	1.8 (2)	C3—C4—C9—C8	−176.72 (13)
N3—N2—C3—C2	−0.6 (2)	N2—N3—C10—C11	179.47 (12)
N3—N2—C3—C4	−179.38 (12)	N3—C10—C11—C16	2.7 (2)
C1—C2—C3—N2	−179.88 (14)	N3—C10—C11—C12	−176.54 (14)
C1—C2—C3—C4	−1.1 (2)	C17—O1—C12—C13	−5.0 (2)
N2—C3—C4—C5	−1.0 (2)	C17—O1—C12—C11	174.34 (14)
C2—C3—C4—C5	−179.75 (14)	C16—C11—C12—O1	−179.20 (13)
N2—C3—C4—C9	177.93 (13)	C10—C11—C12—O1	0.1 (2)
C2—C3—C4—C9	−0.9 (2)	C16—C11—C12—C13	0.2 (2)
C9—C4—C5—C6	−1.1 (2)	C10—C11—C12—C13	179.50 (13)
C3—C4—C5—C6	177.74 (13)	O1—C12—C13—C14	179.53 (14)
C4—C5—C6—C7	−0.8 (2)	C11—C12—C13—C14	0.2 (2)
C5—C6—C7—C8	1.6 (2)	C12—C13—C14—C15	−0.2 (2)
C5—C6—C7—Cl1	−177.40 (11)	C18—O2—C15—C14	3.0 (2)
C6—C7—C8—C9	−0.5 (2)	C18—O2—C15—C16	−176.98 (13)
Cl1—C7—C8—C9	178.51 (11)	C13—C14—C15—O2	179.83 (14)
C1—N1—C9—C8	177.32 (13)	C13—C14—C15—C16	−0.2 (2)
C1—N1—C9—C4	−1.9 (2)	C12—C11—C16—C15	−0.6 (2)
C7—C8—C9—N1	179.30 (13)	C10—C11—C16—C15	−179.88 (13)
C7—C8—C9—C4	−1.4 (2)	O2—C15—C16—C11	−179.42 (13)
C5—C4—C9—N1	−178.56 (13)	C14—C15—C16—C11	0.6 (2)

### Hydrogen-bond geometry (Å, º)

**Table:**

*D*—H···*A*	*D*—H	H···*A*	*D*···*A*	*D*—H···*A*
N2—H2n···N1^i^	0.88 (1)	2.19 (1)	3.0572 (17)	167 (2)

Symmetry code: (i) *x*, −*y*+1/2, *z*−1/2.
